# The Drude‐Smith Equation and Related Equations for the Frequency‐Dependent Electrical Conductivity of Materials: Insight from a Memory Function Formalism

**DOI:** 10.1002/cphc.202100299

**Published:** 2021-07-03

**Authors:** Wei‐Chen Chen, Rudolph A. Marcus

**Affiliations:** ^1^ Noyes Laboratory of Chemical Physics California Institute of Technology 1200 E California Blvd. Pasadena California 91125 USA

**Keywords:** conductivity, Drude equation, Drude-Smith equation, memory function terahertz region

## Abstract

The Drude‐Smith equation is widely used for treating the frequency‐dependent electrical conductivity of materials in the terahertz region. An attractive feature is its sparsity of adjustable parameters. A significant improvement over Drude theory for these materials, the theory includes backscattering of the charge carriers. It has nevertheless been criticized, including by Smith himself, because of the arbitrariness of a step in the derivation. We recall a somewhat similar behavior of back scattering in fluids observed in molecular dynamics computations and discussed in terms of memory functions. We show how theories such as Drude‐Smith and Cocker *et al*. are examples of a broader class of theories by showing how they also arise as particular cases of a memory function formalism that divides the interactions into short and long range.

## Graphical abstract

**Insight into the Drude‐Smith equation** commonly used to treat terahertz electrical conductivity of solids and the role of backscattering is obtained using extensive computer‐based studies of a related quantity, the velocity autocorrelation function of fluids. The latter has been interpreted using a memory function formalism which is then used here to analyze and extend the Drude‐Smith equation.

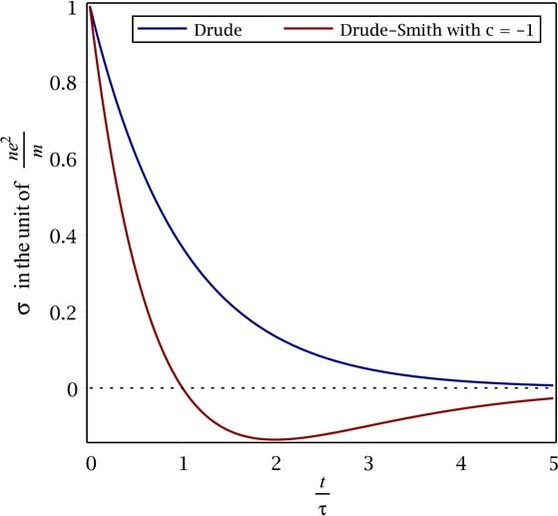

## 1 Introduction

The Drude‐Smith equation for the frequency‐dependent electrical conductivity $\tilde{\sigma }\left(\omega \right)$ was first derived for the conductivity of the liquid metals^[1]^ to treat the backscattering of the charged carriers. It was later applied to semiconductors^[2]^ and is now widely used for treating the frequency‐dependent conductivity of materials in the terahertz regime, e. g. Ref. [3–10]. One of its attractive features is its sparsity of parameters, of which there are two, a Drude‐type relaxation time and a backscattering probability.

We recall that in the original Drude equation for the conductivity there is a relaxation time arising from the interaction of each charge with the other charges. In his extension of the Drude equation, Smith incorporated backscattering and neglected all backscattering collisions after the first one. He noted that “the justification for this procedure is not too clear.”^[1]^ Similar criticism has been made by a number of authors, e. g. Ref. [10–14]. A recent review of THz studies is given in Ref. [14].

**Figure 1:**
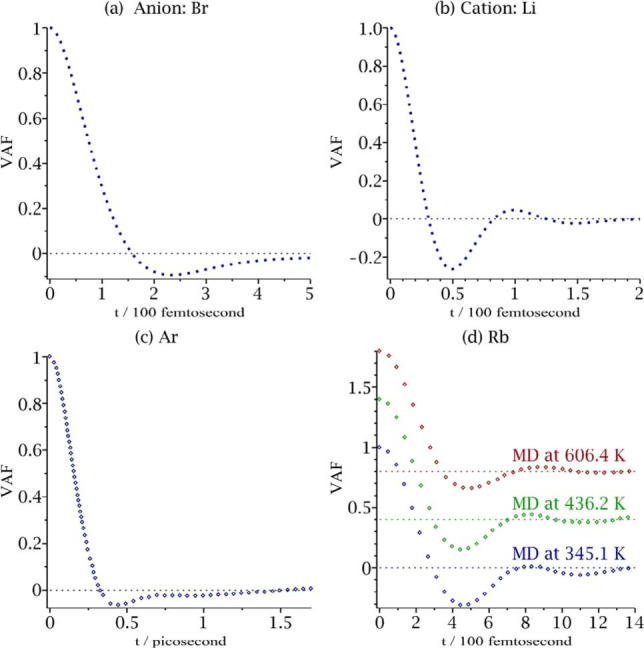
The VAF (dots) in various liquids obtained from molecular dynamics simulations. The (a) Br anion and (b) Li cation in liquid LiBr^[15]^ (c) Liquid Ar^[16]^ (d) Liquid Rb at three temperatures.^[17]^ The horizontal dotted lines indicate zeros in the VAF at each MD simulation.

For a system of free charges the frequency dependent conductivity is related to the velocity autocorrelation function (VAF) by a simple factor and it is useful to recall some numerical results on the VAF for fluids that sheds some light on the behavior of charges in semiconductor conductivity. In particular, backscattering plays a prominent role in the statistical mechanical theory of diffusion in fluids. Examples in the computer‐calculated VAF are given in Figure 1,[^15^, ^16^, ^17^] where either only a single backscattering is seen (for the “collisionally soft” bromide anion and for liquid argon atoms), and a few but small subsequent backscattering events are seen for a collisionally “hard” small cation, such as the Lithium cation or Rubidium, using the hard‐soft concept taken from inorganic chemistry.^[18]^

Computer‐calculated velocity autocorrelation functions (VAF) associated with diffusion are typically interpreted using a memory function, e. g. Ref. [19], In the time domain the VAF often has a small negative contribution, directly showing backscattering. Using the memory function approach^[11]^ in the literature for the conductivity we find that the Cocker *et al*. current autocorrelation function is also included in the present analysis. Of particular interest, this latter equation treats grain boundary scattering, with a Drude term having one lifetime and the scattering at a grain boundary having another lifetime.^[11]^ The memory function approach also suggests a simple extension of the Drude‐Smith theory.

The overall qualitative similarity of the velocity autocorrelation (VAF) plots of fluids, such as that in Figure 1, and the Drude‐Smith plot for the time‐dependent electrical conductivity σ(*t*) of electrons and holes given later in Figure 3(a), and the frequent use of memory functions to treat the former prompted the present treatment. Although the two scattering cross‐sections differ in detail they have some features in common discussed later.

An outline of the paper is as follows: 1) the Drude‐Smith equation is given in the next section. 2) A memory function formalism is introduced and used there to obtain an equation for the complex‐valued conductivity $\tilde{\sigma }\left(\omega \right)$ . It is shown there how the Drude‐Smith equation and the Cocker *et al*. equation arise as special cases of that formalism. 3) The relation to the velocity autocorrelation function for diffusion in fluids, extensively studied via computations in the literature, is discussed together with some results resulting from the latter. 4) Various experimental results on terahertz conductivity are considered and discussed. 5) Concluding remarks are given in the last section.

## 2 Frequency‐Dependent Electrical Conductivity, Autocorrelation Function and Drude‐Smith

We consider the component *j*(*t*) of the electric current along the direction of the electric field. We recall that the electric current autocorrelation function is related to the frequency‐dependent conductivity per unit volume $\tilde{\sigma }\left(\omega \right)$ by the Fourier‐Laplace transform[^20^, ^21^, ^22^],

(1) $$\tilde{\sigma }\left(\omega \right)=\frac{1}{{Vk}_{B}T}\int_{0}^{\infty }exp\left(i\omega t\right)\left\langle j\left(0\right)j\left(t\right)\right\rangle dt.$$

The inverse relation is given by

(2) $$\left\langle j\left(0\right)j\left(t\right)\right\rangle =\frac{{Vk}_{B}T}{2\pi }\int_{-\infty }^{\infty }exp\left(-i\omega t\right)\tilde{\sigma }\left(\omega \right)d\omega .$$

Each *j* in Equation (2) involves a sum over the particles. In this double sum we neglect cross terms from different particles, and thereby the product of *j's* in Equation (1) is proportional to *N*, the number of particles. As in the Drude‐Smith equation we consider the contribution of one kind of particle. We recall that the Drude‐Smith equation for the frequency‐dependent conductivity $\tilde{\sigma }\left(\omega \right)$ is given by:[^1^, ^2^]

(3) $$\tilde{\sigma }\left(\omega \right)=\frac{ne^{2}\tau }{m}\left(\frac{1}{1-i\omega \tau }+\frac{c}{{\left(1-i\omega \tau \right)}^{2}}\right),$$

where *n*, *e*, *m* and τ are the particle density, charge, effective mass and lifetime, respectively, and *c* is a constant lying in the interval (0,‐1). From Equations (2) and (3) using contour integration to evaluate the integral we also obtain the Drude‐Smith equation [Eq. (4)] in the time domain.

(4) $$\left\langle j\left(0\right)j\left(t\right)\right\rangle =\frac{Ne^{2}}{m}\left(1+\frac{ct}{\tau }\right)exp\left(\frac{-t}{\tau }\right).$$

## 3 Memory Function Formalism and Application to $\tilde{\sigma }\left(\omega \right)$

### 3.1 General

We consider next the generalized Langevin equation^[23]^ for the velocity of the charged particle *v*(*t*), containing a memory kernel M(*t*) and given by Equation 5:^[24]^

(5) $$\frac{dv\left(t\right)}{dt}=-\int_{0}^{t}M\left(t-t_{1}\right)v\left(t_{1}\right)dt_{1}+R\left(t\right),$$

where *R*(*t*) is a random force. Multiplying, as usual, by *v*(0), taking an ensemble average, and noting that *v*(0) and *R*(*t*) are uncorrelated and so ⟨*v*(0)*R*(*t*)⟩=⟨*v*(0)⟩⟨*R*(*t*)⟩, we have Equation 6:

(6) $$\left\langle v\left(0\right)\frac{dv\left(t\right)}{dt}\right\rangle =\int_{0}^{t}M\left(t-\tau \right)\left\langle v\left(0\right)v\left(\tau \right)\right\rangle d\tau .$$

Taking the Fourier‐Laplace transform of this equation and integrating by parts yields the velocity autocorrelation function in frequency space $\tilde{C}\left(\omega \right)$ [Eq. 7]:[^20^, ^21^, ^22^]

(7) $$\tilde{C}\left(\omega \right)=\int_{0}^{\infty }\exp\left(i\omega t\right)\left\langle v\left(0\right)v\left(t\right)\right\rangle dt=\frac{\left\langle v^{2}\left(0\right)\right\rangle }{\tilde{M}\left(\omega \right)-i\omega },$$

where $\tilde{M}\left(\omega \right)$ is the Fourier‐Laplace transform of M(*t*).

For the specific conductivity $\tilde{\sigma }\left(\omega \right)$ we note its relation to the velocity autocorrelation function [Eq. 8]^[20]^

(8) $$\tilde{\sigma }\left(\omega \right)=\frac{ne^{2}}{k_{B}T}\int_{0}^{\infty }exp\left(i\omega t\right)\left\langle v\left(0\right)v\left(t\right)\right\rangle dt,$$

where we used Equation (1), neglected the cross‐terms in the double sum over the particles in the product of the *j’*s, and note that [Eq. 9]

(9) $$j\left(t\right)=N\;e\;v\left(t\right),$$

where *v*(*t*) is the component of the velocity of the charge carrier along the direction of the electric field. From Equations (7) and (8) we have [Eq. 10]

(10) $$\tilde{\sigma }\left(\omega \right)=\frac{ne^{2}}{m}\frac{1}{\tilde{M}\left(\omega \right)-i\omega }.$$

We turn next to an expression for $\tilde{M}\left(\omega \right)$ , confining our attention initially to models that build on the Drude model, Smith's being one example. This memory function has to be such that it yields the Drude model in the appropriate limit. The Drude model contains no time constant associated with the build‐up, after an electric field pulse, of the velocity distribution of the charged particle. The associated memory term that yields this instantaneous build‐up is therefore a δ‐function, one defined for the time domain *t*≥0. A second term in a suitable memory function is associated with the backscattering.

Models for M(*t*) for molecules in fluids that involve backscattering include a sum of two exponentials,^[25]^ a sum of a Gaussian and an exponential,^[26]^ and others. The term in the memory function associated with a small lifetime (in our case a delta function) reflects the instantaneous phase space redistribution of the other charges that accommodate themselves to interact with the charged carrier (the Drude term) after an electric field pulse. The term in the memory function associated with shorter range interactions (backscattering) has instead a long lifetime.

### 3.2 Application of Memory Function to Specific Models for $\tilde{\sigma }\left(\omega \right)$

Common two‐term memory functions M(*t*) in the literature have four parameters: the two amplitudes and the two lifetimes.[^25^, ^26^] If we choose one of the memory function terms to be a delta like function, we are left with three parameters in M(*t*), which we write as *p*, *q*, and *r*:

(11) $$M\left(t\right)=p\;\delta^{+}\left(t\right)+q\;exp\left(-rt\right)$$

with *M*(*t*)=0 for *t*<0. In Equation (11) δ^+^ (t) is a Dirac delta‐like function for the interval (0, ∞), instead of (−∞, ∞), i. e., $\int_{0}^{\infty }f\left(t\right)\delta^{+}\left(t\right)dt=f\left(0\right)$ . (It is also the derivative of a unit step function at *t*=0.) The corresponding conductivity $\tilde{\sigma }\left(\omega \right)$ in frequency space, obtained using Equation (11), is

(12) $$\tilde{\sigma }\left(\omega \right)=\frac{ne^{2}}{m}\frac{1}{p+\frac{q}{r-i\omega }-i\omega }.$$

Equation (12) can be rewritten as

(13) $$\tilde{\sigma }\left(\omega \right)=\frac{ne^{2}}{m}\frac{\left(r-i\omega \right)}{\left(i\omega -\omega_{+}\right)\left(i\omega -\omega_{-}\right)},$$

where

(14) $$2\omega_{\pm }=p+r\pm \sqrt{s},$$

and *s*, the discriminant, is given by

(15) $$s={\left(p-r\right)}^{2}-4q.$$

The correlation function C(*t*) associated with the current *j*(*t*) is given by Equation (2), the inverse of the Fourier‐Laplace transform of $\tilde{\sigma }\left(\omega \right)$ . In Equation (2) we form a contour integral by completing the contour by a large semicircle in the lower half of the complex ω plane and so obtain Equation 16

(16) $$\left\langle j\left(0\right)j\left(t\right)\right\rangle =\frac{Vk_{B}T}{2\pi i}\oint exp\left(-i\omega t\right)\tilde{\sigma }\left(\omega \right)d\omega .$$

Applying Cauchy's residue theorem using Equations (13)–(15), one obtains for the electric current autocorrelation function ⟨*j*(0)*j*(*t*)⟩

(17) $$\left\langle j\left(0\right)j\left(t\right)\right\rangle =\frac{e^{2}k_{B}T}{m}\frac{\left(\omega_{+}-r\right)e^{-\omega_{+}t}+\left(r-\omega_{-}\right)e^{-\omega_{-}t}}{\left(\omega_{+}-\omega_{-}\right)}.$$

There are three types of behavior for the autocorrelation function given by Equation (17), depending on whether $\omega_{\pm }$ in Equation (14) has *s*>0, *s*<0, or *s*=0. When *s*>0, the roots $\omega_{+}$ and $\omega_{-}$ are real and so the autocorrelation function decays as a double exponential (a biexponential). When *s*<0, the $\omega_{+}$ and $\omega_{-}$ are complex‐valued and it decays instead in an oscillating manner. When *s*=0, the two poles arising from Equations (13)–(15) coalesce and the double root $\omega_{\pm }=\left(p+r\right)/2$ contributes to the contour integral. The current autocorrelation function then becomes Equation 18

(18) $$\left\langle j\left(0\right)j\left(t\right)\right\rangle =\frac{e^{2}k_{B}T}{m}\left(1+\frac{\left(r-p\right)t}{2}\right)\exp\left(-\frac{\left(p+r\right)t}{2}\right).$$

This equation has only two constants *p* and *r*, and the equation is equivalent to the Drude‐Smith Equation (4), the two constants being related to the *c* and τ in the latter by (r+p)/2=1/τ and (r-p)/2=c/τ .

Another special case of Equation (17) is the underdamped case. (*i. e*., *s*<0). In this case the equation for the electrical current autocorrelation function is that of an exponentially damped periodic decay [Equation 19]:

(19) $$\left\langle j\left(0\right)j\left(t\right)\right\rangle =\frac{e^{2}k_{B}Te^{-\frac{1}{2}\left(p+r\right)t}}{m}\left[\left(\frac{r-p}{\left|\sqrt{s}\right|}\right)\sin\left(\frac{\left|\sqrt{s}\right|t}{2}\right)+\cos\left(\frac{\left|\sqrt{s}\right|t}{2}\right)\right].$$

### 3.3 Application to the Corker *et al*.^[11]^ Scattering Equation for $\tilde{\sigma }\left(\omega \right)$

Corker *et al*. developed a treatment that includes scattering from grain boundaries and the probability of crossing them.^[11]^ Their equation for $\tilde{\sigma }\left(\omega \right)$ can be written as Equation (20) (eq 47 in Ref. [11])

(20) $$\tilde{\sigma }\left(\omega \right)=\frac{ne^{2}}{m}\frac{\tau }{1-i\omega \tau }\left(1+\frac{c\left(R\right)}{1-i\omega /a}\right),$$

partly in their notation, where *n* and *τ* denote their *N* and *τ*’. One sees that their model for $\tilde{\sigma }\left(\omega \right)$ has two poles on the negative imaginary axis, at ‐*i*/*τ* and at ‐*ia*. From the inverse transform obtained by contour integration over the lower half plane one sees therefore that the current autocorrelation function ⟨*j*(0)*j*(*t*)⟩ of Corker *et al*.^[11]^ has a biexponential decay, with decay times of *τ* and 1/*a* and no oscillations or half oscillations. The factor *c*(*R*) depends exponentially on the grain size.^[11]^ If the two poles merged it would reduce to the Drude‐Smith expression, but with a grain boundary interpretation for the non‐Drude term.

## 4 Comparison with the Velocity Autocorrelation Function of Fluids

To gain additional insight into the Drude‐Smith and related equations we compare with some of the many computer‐based results on the velocity autocorrelation function of fluids with the results on semiconductors. In marked contrast to the current autocorrelation function of semiconductors almost all the studies of the velocity autocorrelation function in fluids, with a few rare exceptions,[^27^, ^28^] have been made by computer simulations rather than in laboratory experiments. The computed velocity autocorrelation functions (VAF) are typically plotted versus time, and so immediately revealed visually any backscattering, as in Figures 1(b) and 1(d) given earlier. However, observing such detailed temporal data would require an optical rather than a THZ spectrum ‐ judging from the τ values ∼10 fs inferred from experiment (e. g., in a Ref. [44] cited later).

The molecular dynamics (MD) simulation of molten LiBr and other salts was studied by Lantelme *et al*.^[15]^ and some of their results were given in Figure 1. To treat the data they introduced a memory function that is a sum of two exponential terms, and so is a more general function than the present sum of a delta function and an exponential. The resulting correlation function has three exponential terms and thereby as many as 6 parameters if none of the roots coincide, instead of only two parameters in the Drude‐Smith expression The functions fitted well their computer simulation results, as seen in the Figures in Ref. [15].

The MD calculations also bring out, as noted earlier, the concept of hard and soft acids and bases drawn from inorganic chemistry.^[18]^ Because a cation is typically “harder” than an anion in its contact with other particles, the VAF of a cation tends to have oscillations,[^29^, ^30^, ^31^] a deviation from the Drude‐Smith equation.

## 5 Discussion

### 5.1 Drude versus Drude‐Smith Equations

**Figure 2:**
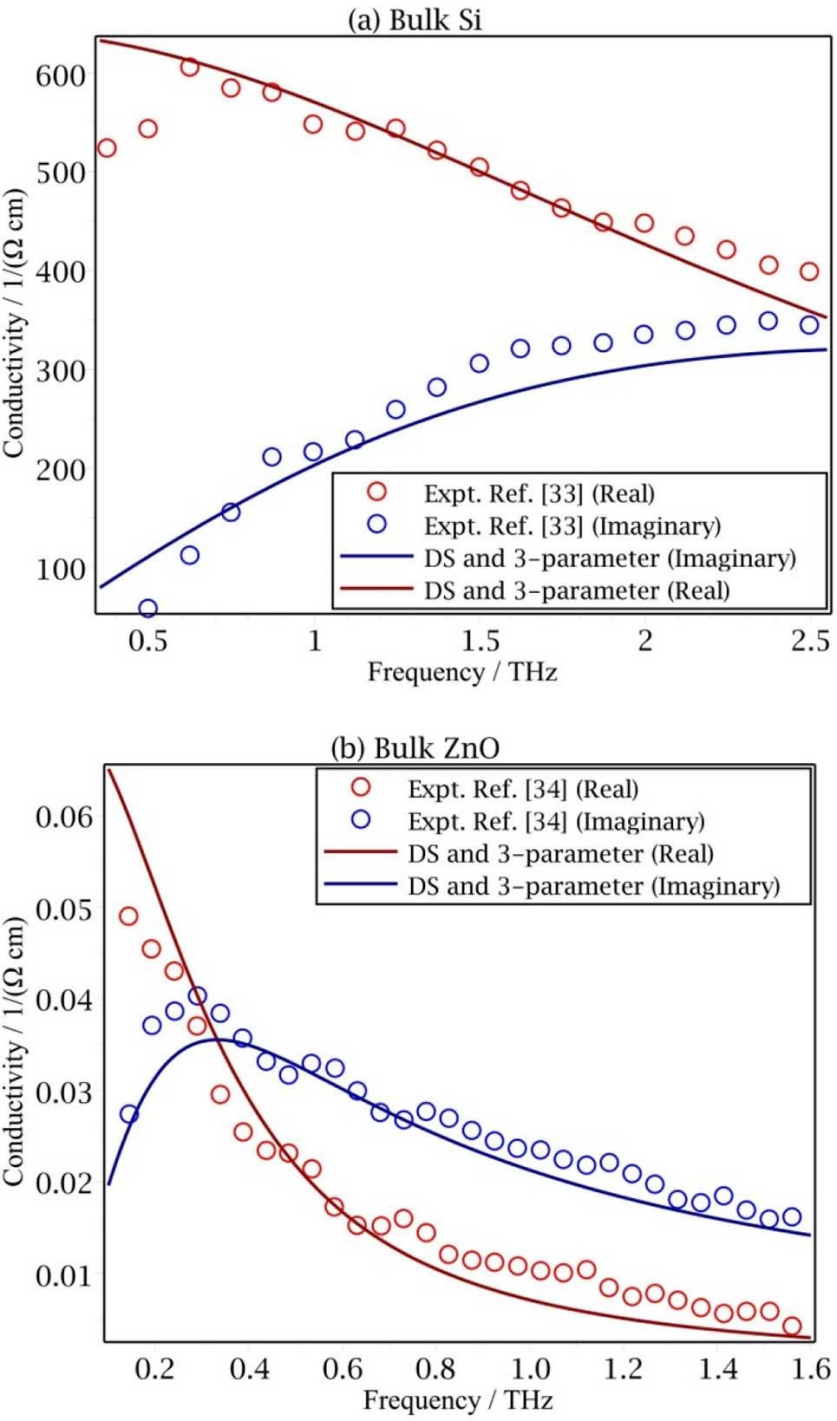
Frequency dependent photoconductivity of bulk (a) silicon^[33]^ and (b) ZnO.^[34]^ The lines are the best fitted result of the Drude‐Smith formula, Eq. (5) and 3‐parameter memory kernel conductivity, Eq. (12). Both fits give overlapping results and are similar to the Drude formula.

The Drude equation, $\tilde{\sigma }\left(\omega \right)$ =*ne* ^2^ *τ*/*m*(1‐*iωτ*) is known to successfully describe the frequency dependence of the conductivity in a most metals.^[32]^ The photoconductivity in a defect‐free bulk semiconductor is also expected to follow the Drude equation, since backscattering is expected to be rare. For example, Titova *et al*. and Baxter *et al*. applied time‐resolved terahertz spectroscopy to bulk silicon^[33]^ and ZnO,^[34]^ respectively. The data are reasonably well described by the Drude current autocorrelation function, an equation with no backscattering. Both fitted results of the Drude‐Smith formula, Eq. (5), and 3‐parameter memory kernel conductivity, Eq. (12), as shown in Figure 2, and are similar to the results of the Drude formula.

When there is significant backscattering, as in nanoparticle semiconductors, the Drude‐Smith equation is, as noted earlier, widely used for fitting experimental data on the frequency‐dependent conductivity in the terahertz region. The Drude equation gives a monotonic decay in time, while the Drude‐Smith equation corresponds to the “critically damped” case, that has a minimum and slowly approaches to zero at long times with no oscillatory behavior, as discussed in the previous section. The plot versus time also permits a direct comparison with extensive data on molecular dynamics computations of the velocity autocorrelation function (VAF) in fluids, which are typically plotted in time space. The back‐scattering feature, for example, is immediately seen visually in plots of the VAF versus time, e. g., Figures 1(b) and 1(d).

**Figure 3:**
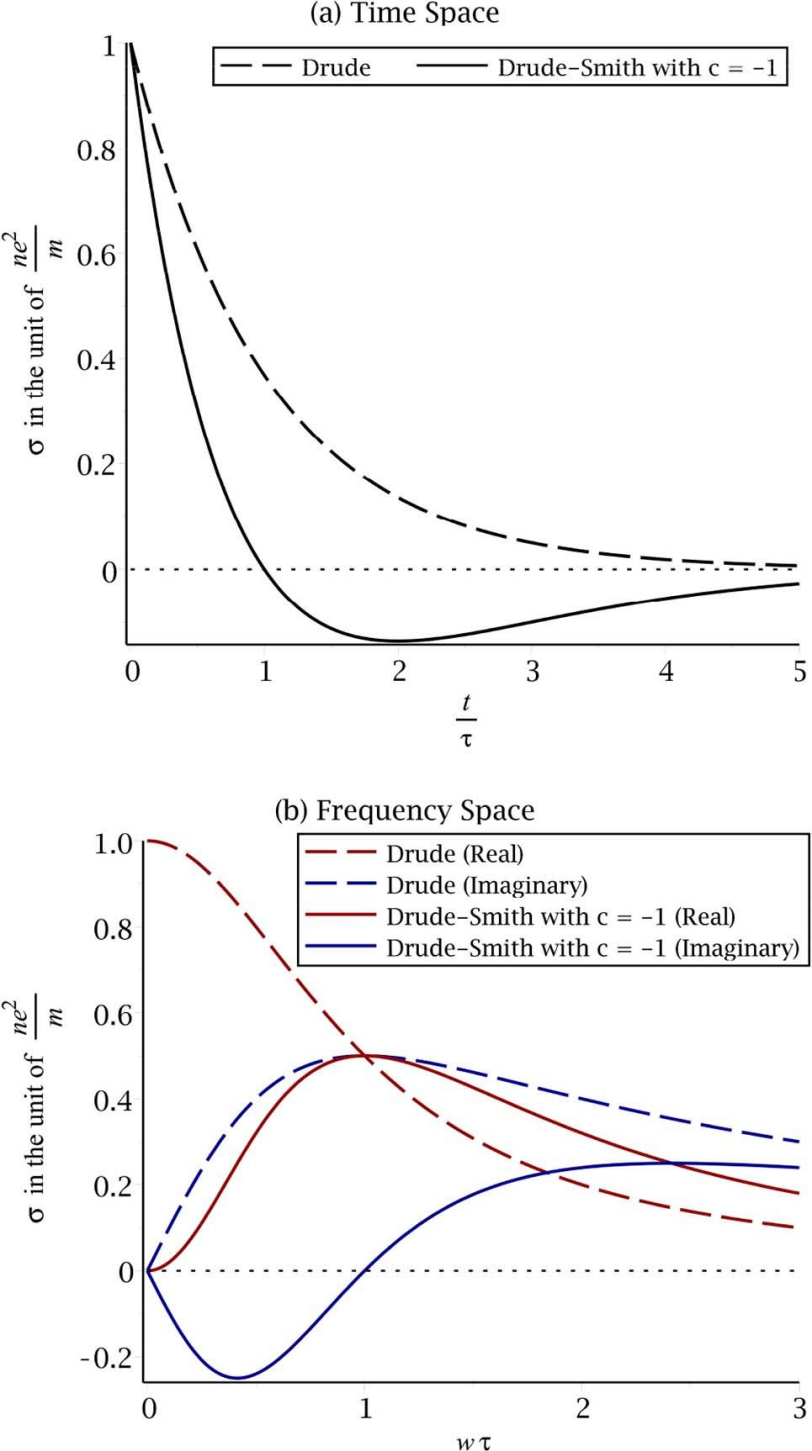
Comparison of the Drude and Drude‐Smith (with c=−1) equations in (a) time and (b) frequency space. The dashed and solid lines are the Drude and Drude‐Smith equations, respectively.

The corresponding difference between Drude and Drude‐Smith equations in frequency spaces is shown in Figure 3. For the Drude‐Smith equation the imaginary part is negative in the low frequency region, and then becomes positive as the frequency increases. In the terahertz experiments the latter feature may not be easily observed because of the limits of the spectral range used in those experiments.

### 5.2 Comparison with Terahertz Data

**Figure 4:**
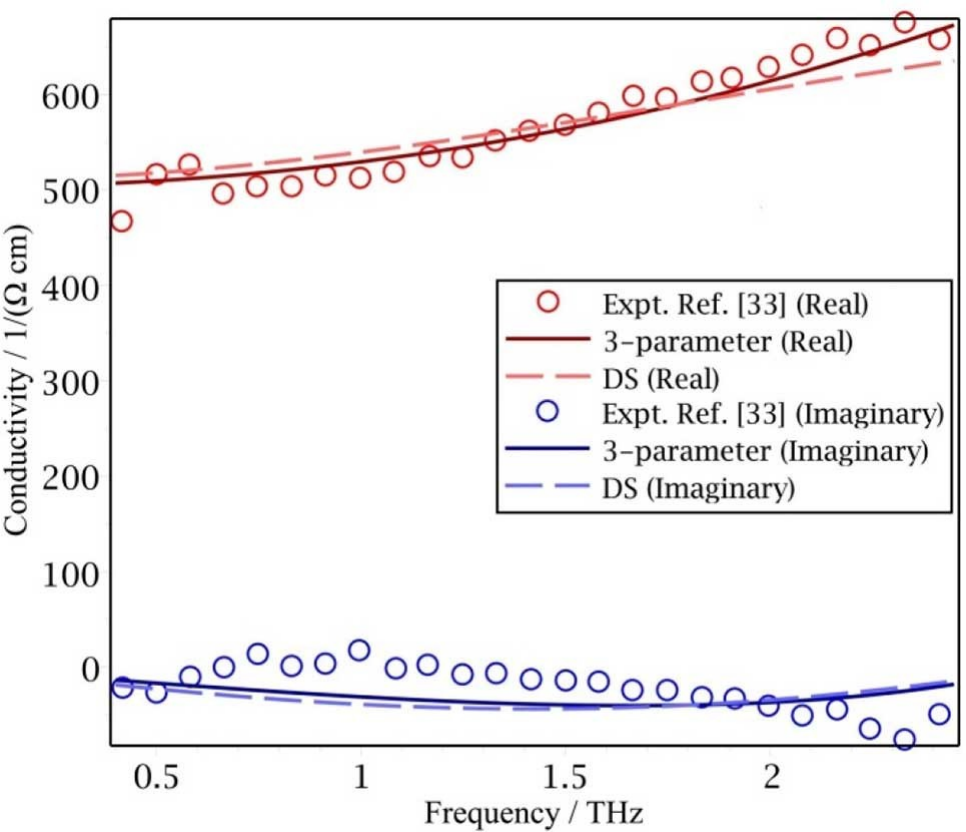
The frequency dependent photoconductivity of a silicon nanocrystal film (SiO_0.4_) at 10 ps after photoexcitation.^[33]^ The 1/*r* in the fitted unconstrained memory kernel conductivity equals 0.24 THz, which is much shorter than the region of most experimental measurements.

When in Eq. (11) *r*≫1 THz, the corresponding lifetime in the exponential term is much shorter than a picosecond. In terahertz experiments the study of the dynamics is typically around the picosecond region. An exponential function with a very fast decay and a delta function give a very similar contribution in the memory formalism. In this extreme of using a delta function, the fit using the 3‐parameter memory kernel gives a behavior very similar to the Drude‐Smith result, as seen in Figure 4 and so here this 3‐parameter memory function formalism gives little improvement over the Drude‐Smith result.

**Figure 5:**
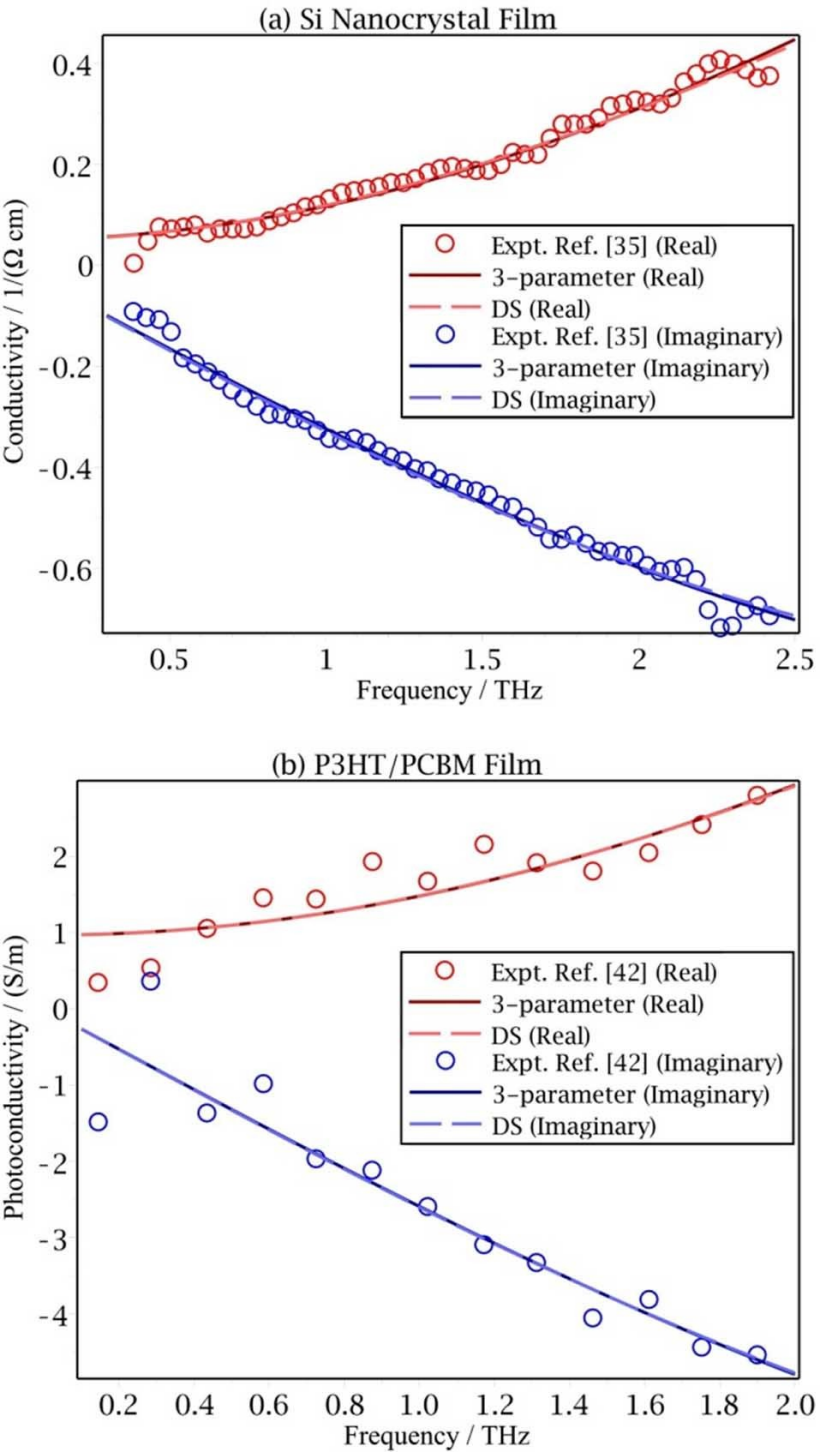
The Drude‐Smith equation and Equation (12) are very similar in fitting experiments with nearly perfect velocity reversal. (a) Silicon nanocrystal film measured at 1100 °C.^[35]^ The fitted *c* value in Equation (12) is −0.985. (b) A blend of Regioregular poly(3‐hexylthiophene) and [6,6]‐phenyl‐C61‐butyric acid methyl ester (P3HT/PCBM) film excited at 800 nm.^[42]^ The fitted c value is −0.967.

We note that in the Drude‐Smith formula a very negative *c* value (*c*≈−1) correspond to a complete velocity reversal on the first collision. Experiments often give a *c* value between −0.6 and −0.9, as in Table 1. In the case of granular boundaries in nanoparticles the velocity of a charged particle can be easily reversed in collision. Some materials give nearly perfect velocity reversal (−1≤*c*≤−0.95).[^35^, ^42^] The Drude‐Smith equation and Eq. (12) then gave nearly overlapping results in fitting these experiments, as seen in Figure 5.

**Table 1:** The range of fitted *c* values, obtained by fitting the Drude‐Smith equation to the conductivity data.

Material	*c* values
Si nanocrystal films^[33]^	−0.61–−0.995
Si nanocrystalline^[35]^	−0.98–−0.998
VO_2_ nanocrystal films^[36]^	−0.59–−0.71_
W‐doped VO_2_ nanocrystal films^[37]^	−0.62–−0.68_
SnO_2_ nanowires^[38]^	−0.90–−0.94_
ZnO[^39^, ^40^]	−0.68–−0.92_
CdSSe nanobelts^[41]^	−0.79–−0.96_
TiO2 nanoparticles^[40]^	−0.87_

Regarding the criticism[^11^, ^12^, ^13^, ^14^] that the Drude‐Smith formulation does not include back scattering terms after the first collision, it is useful to recall that a scattering event is three‐dimensional and not the one‐dimensional model sometimes used, and what counts after the collision is the change in the component of the velocity along the direction of the initial velocity. That component can be quite small when the differential scattering cross‐section is substantial over a wide range of scattering angles. Any further collisions involve a similar distribution of scattering angles and a convolution leads to, particularly in the case of soft colliders, very little final component of the velocity along the initial direction. This situation is not too different visually from the Drude‐Smith like behavior and in this sense a conducting electron can be regarded as a soft collider. The behavior that the “soft” fluid and the scattered electron share is that their scattering is three‐dimensional rather than one‐dimensional and that both appear to interact as soft particles.

We note that the VAF of fluids and the electrical conductance of semiconductors have some features in common, perhaps explaining the approximate similarity of their VAF's. Both have a thermal distribution of velocities in a reflecting system that, on averaging over the velocities, causes a rapid initial decrease in a plot of the VAF versus time plot. They also may have in common a “cage effect”, well known in the chemistry literature for molecules in fluids, and in disordered solids there may be a “rattling” of the charged particle in a cage created by grain boundaries. Such a rattling is described by the damped periodic function for the underdamped regime, as in the present Eq. (19).

There is a shortcoming in using in the memory function a δ function. Because of a property (stationarity) of the velocity autocorrelation function one can show that *C(t)*=*C(‐t)* and hence that d*C(t)*/d*t* a*t t*=0. This property is clearly not obeyed by the Drude or Drude‐Smith *C(t)*’s or indeed, one can show analytically, is not obeyed by a memory function which the sum of two simple exponentials and so they too will fail at small *t* and hence at sufficiently high frequencies. A typical τ appearing in eq shorter than this. There are experiments on the frequency‐dependent electrical conductivity that have been performed at higher frequencies in the THz range, e. g., as in plots of $\tilde{\sigma }\left(\omega \right)$ σ(ω) up,^[34]^ to ∼13 THz in [44] and up to ∼20 THz in [45], and so much higher than the frequencies appearing in the plots here, ∼2 THz. The Drude and Drude‐Smith equations appear to adequately represent the experimental data in those regions. In passing we note that Smith first obtained the Drude‐Smith equation for the conductivity of liquid metals at optical frequencies of several eV,^[1]^ ∼700 THz. It fitted the data very well and has a detailed structure that one can't see in the THz spectral region.

We comment further on events happening during the short time after an applied electric field has been introduced and approximated by the use of a δ‐function in the memory function. Just before *t*=0 there is no applied electric field and so just after the introduction of an external electric field the distribution of charges and velocities of the charges around a charge carrier is that for zero applied field and so the motion of the carrier is ballistic. The use of the δ‐function in Eq. (11) ignores what happened during this short time period. Shortly after *t*=0 the distribution of charges and velocities around the carrier has become a perturbed distribution whose deviation from the equilibrium distribution is proportional to the applied electric field. Mathematically, *M*(*t)* is an even function of *t* at short times.^[19]^ The use of a δ‐function in *M*(*t)*, or indeed of any function which at small times is not an even function of *t*, simple exponential decay, for example, does not satisfy this condition, though one can select a function that does, e. g., Ref. [26].

When there is an “optically active” low frequency phonon in the sample in the terahertz region it too can contribute to the terahertz spectrum.^[43]^ It is related to a dipole correlation function, rather than to the electric current correlation function for electrons or holes. Its $\tilde{\sigma }\left(\omega \right)$ is typically a Lorentzian (bound oscillator) and is not considered here. We also note that we do not treat plasmons here. They obey the Drude‐Smith Eq. (3) with *c*=−1, the other symbols having somewhat different physical significance.^[3]^

Eq (5) presumes that there is one type of charged carrier. When both holes and electrons are present one limiting situation is that the hole and the electron are tightly coupled, so forming an exciton. The other limit is that they move independently of each other. In that case the $\tilde{\sigma }\left(\omega \right)$ is the sum of contributions from each type of carriers and their contributions have sometimes been resolved. A recent contact‐free method is cited later in [53], which also makes a comparison with earlier results.

### 5.3 Memory Functions

Memory functions used for treating the statistical temporal behavior of dynamical quantities can be introduced either via the time‐evolution of a dynamical quantity using a generalized Langevin equation, e. g. Ref. [20, 46], or via the evolution of the probability distribution function of that quantity using a generalized master equation, e. g. Ref. [47, 48], the relation between the two approaches being described in Ref. [20, 46]. We have used the former, prompted by its extensive application in interpreting molecular dynamics computations of the velocity autocorrelation function, e. g. Ref. [20]. Kenkre and coworkers in their study of effective medium theories used instead a master equation with a memory function of the form given by .Eq. (11), e. g. Ref. [47] and chapter 13 in Ref. [48]. We also note that effective medium theories for treating frequency‐dependent electrical conductivity have been discussed in terms of memory functions using a generalized master equation for the time‐dependent probability distribution of position on lattices, e. g. Ref. [47–49]. A problem involving spatial disorder has thereby been translated into one involving temporal behavior, the longer times corresponding to longer distances. A model describing the Drude‐Smith equation in terms of broadened resonances for motion in a confined region has been given in Ref. [50].

A pioneering use of a memory function for a velocity autocorrelation function was given by Berne *et al*.,^[51]^ who used the memory function in Eq. (11) with p=0, thereby restricting the number of parameters to two instead of three and so it representing a special case of the present expression.

For comparison with Smith's method we note that that in general there are several different methods for treating frequency‐dependent coefficients of dynamical variables using nonequilibrium statistical mechanics. One of these is the method used by Smith, using a theory for the response of the system to a unit electric pulse and taking its Fourier‐Laplace transform.^[1]^ A second is the linear response theory which expresses the frequency‐dependent dynamical coefficient in terms of a current autocorrelation function, the method used in the present article. A third method is the use of an external electric field and turning it off at any given time and studying the response[^20^, ^52^] All three methods give the same result.

## 6 Conclusions

The Drude‐Smith equation, widely used in terahertz conductivity studies of materials, is shown to be a special case of a more general formulation treated using a memory function. In this formalism the Drude‐Smith equation emerges as the “critically damped” case, with only two parameters. The behavior is related to that of a velocity autocorrelation function of fluids consisting of ”soft” particles such as anions and large rare gas atoms. An expression given by Cocker *et al*.^[11]^ that has two parameters and is intended to treat systems with grain boundaries, is another special case in this memory function formalism. The formalism offers a systematic way of extending the treatment to other frequencies in the frequency‐dependent electrical conductivity spectrum.

## Conflict of interest

The authors declare no conflict of interest.
